# The Application of Optical Coherence Tomography in Musculoskeletal Disease

**DOI:** 10.1155/2013/563268

**Published:** 2013-01-15

**Authors:** Christopher Rashidifard, Christopher Vercollone, Scott Martin, Bin Liu, Mark E. Brezinski

**Affiliations:** ^1^Center for Optical Coherence Tomography and Optical Physics, Department of Orthopedic Surgery, Brigham and Women's Hospital, 75 Francis Street, Boston, MA 02115, USA; ^2^Harvard Medical School, 250 Longwood Avenue, Boston, MA 02115, USA

## Abstract

Many musculoskeletal disorders (MDs) are associated with irreversible bone and cartilage damage; this is particularly true for osteoarthritis (OA). Therefore, a clinical need exists for modalities which can detect OA and other MDs at early stages. Optical coherence tomography (OCT) is an infrared-based imaging, currently FDA approved in cardiology and ophthalmology, which has a resolution greater than 10 microns and acquisition rate of 120 frames/second. It has shown feasibility for imaging early OA, identifying changes prior to cartilage thinning both in vitro and in vivo in patients and in OA animal models. In addition, OCT has shown an ability to identify early rheumatoid arthritis (RA) and guide tendon repair, but has the potential for an even greater impact. Clinical trials in OA are currently underway, as well as in several other MDs.

## 1. Introduction 

Musculoskeletal diseases are one of the leading causes of disability in the United States. Fifty million adults in USA have been diagnosed with arthritis, rheumatoid arthritis, gout, systemic lupus erythematosus, or fibromyalgia, and approximately 1 in 3 people between ages 18 to 64 with diagnosed arthritis have work limitations [1, 2]. Similarly, disease of the periarticular structures, such as tendons and ligaments, contributes to additional disabilities and limitations. Full- or partial-thickness tears of rotator cuff tendons (RCT) are relatively common; they occur in approximately 30% of the population and represent around 4.5 million clinic visits and 40,000 surgeries in the USA per year [3]. Arthritis also affects the pediatric population, with an estimate 294,000 children under the age of 18, or 1 in every 250, having some form of arthritic or rheumatologic condition [4]. Studies estimate that by the year 2030, 67 million Americans older than 18 years will have doctor-diagnosed arthritis [5]. 

This paper examines the potential of the new micron-scale imaging technology and optical coherence tomography (OCT), for the management of musculoskeletal disease. It focuses on the existing clinical need for a high-resolution micron-scale imaging system in the field of orthopedics, including osteoarthritis (OA), rheumatoid arthritis (RA), and rotator cuff repair (RCR). However, this is far from the full extent of potential applications. Early detection of disease, understanding early disease markers, and accurate assessment of tissue microstructure are necessary to increase success of treatment, reduce patient morbidity, and determine the progress of future therapeutics in hopes of improving patient outcomes. 

## 2. The Developments and Advantages of OCT Nontransparent Tissue Imaging

Based on technology from telecommunications, optical coherence tomography (OCT) systems have been modified to accommodate many medical specialties, ranging from ophthalmology and cardiology to orthopedics. The first two applications are FDA approved and are routinely used in patients. Since 1994 our lab has been developing OCT for nontransparent tissue, from engineering and physics to clinical trials.

Analogous to ultrasound, OCT is an imaging modality that utilizes infrared light rather than sound. As infrared light is generated, it is split into both a sample and reference arm. Light reflecting back from the sample combines with light from the precisely controlled reference arm mirror. The intensity of interference is measured using a technique called low-coherence interferometry, with this intensity plotted as a function of tissue depth as the reference arm mirror is moved. As the beam is scanned across the surface of the tissue, two and three dimensional data sets are derived [14]. These image sets can then be used to interpret the microstructure of the tissue.

**Figure 1:**
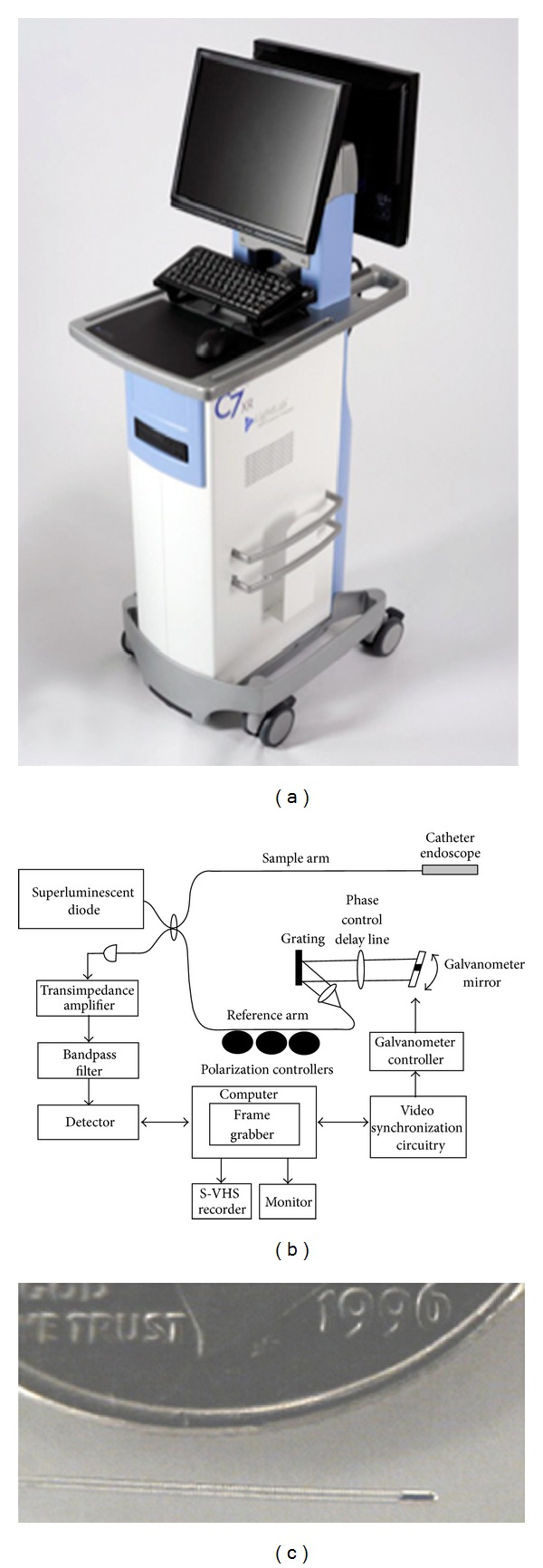
Image (a), schematics (b), and catheter (c) of LightLab OCT imaging engine. Courtesy of LightLab imaging.

Current clinical imaging modalities such as MRI, CT, and X-ray are critical to the diagnosis of disease. However, early disease diagnosis requires micron-scale, real-time imaging. OCT has shown many advantages over current imaging modalities such as a resolution 25x higher than any clinical subsurface imaging modality, a data acquisition speed of 120 high-resolution images per second, and the ability to perform imaging through a 0.017-inch fiber-optic endocatheter with automated probe pull-back [15–17]. These characteristics make OCT an ideal system for the clinical setting in terms of speed and volumes of information gathered on both target and auxiliary structures. Additionally, the OCT engine is comparative in size to a portable ultrasound machine, which allows easy transport into procedure rooms (Figure 1). These advantages make OCT an ideal tool for both the clinical and research settings.

Finally, OCT can be combined with many adjuvant techniques: Doppler OCT, OCT elastography, OCT spectroscopy, second-order correlation (SOC) OCT, and polarization-sensitive OCT (PS-OCT). These adjuvant technologies are described in further detail in the appendix. For the purposes of this paper, we will examine the ability of OCT, focusing primarily on structural OCT and PS-OCT, to identify and track early disease progression and its potential for the early diagnosis of OA, RA, and RCR.

## 3. OCT's Contribution to the Investigation of Joint and Musculoskeletal Disease

### 3.1. In Vitro and In Vivo Human OA Data

The pathogenesis of osteoarthritis (OA) is multifactorial and encompasses interlaced genetic, biochemical, metabolic, and inflammatory components. This complex disease process is generally attributed to one cell, the chondrocyte, which is signaled to increase both proliferation and synthetic and degradative efforts when disrupted [18–23]. The signaling cascade is started by damage to the cartilage, either through a single macrotraumatic event or several microtraumatic events, that fragment and release proteoglycans and type II collagen into the synovial fluid of the joint [24–31]. This may occur either at the surface or the cartilage-subchondral bone interface. Although the chondrocytes react at the site of articular damage and release degradative enzymes in hopes of self-repair, such as metalloproteases, the effort is generally futile [22, 32–34]. These chemical changes in the joint only amplify the amount of damage to the articular cartilage, which reacts abnormally. Under previously normal biomechanics and load characteristics, cartilage is more easily deformed, and fluid loss is increased by the loss of glycosaminoglycans [35, 36]. 

Advanced osteoarthritis is visualized by thickening of subchondral bone and synovium, bone spurs at the joint periphery, and feathering/full thickness defects in the articular surface [37, 38]. However, by this stage of OA advancement, reversal is unlikely. Thus, it is imperative to diagnose OA early. Predictive early changes in an arthritic joint consist of the loss of an organized collagen and glycosaminoglycan matrix, consistent with less water retention in articular cartilage with increased load [7, 39]. Since the OA cascade is so detrimental to the cartilage, it is optimal to diagnose the disease in its early manifestations so that preventative therapies can be initiated.

**Figure 2:**
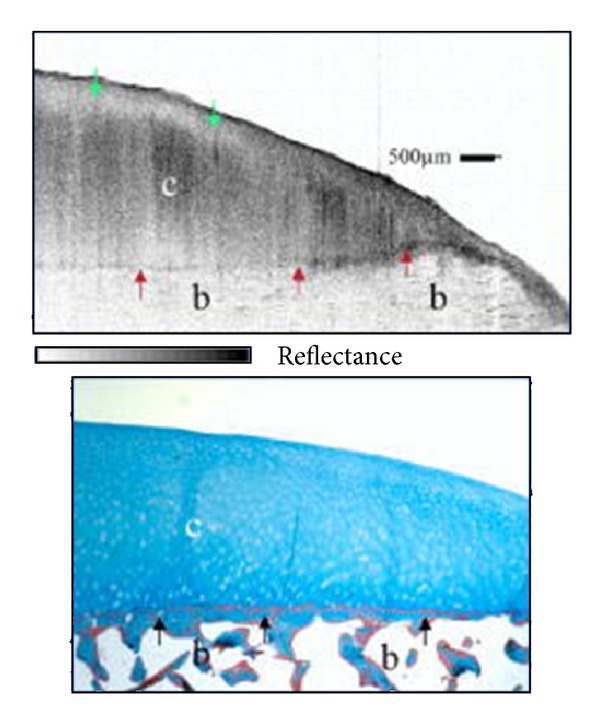
OCT of normal cartilage. The OCT image (top) shows clearly defined bone (b), cartilage (c), and cartilage/bone interface (red arrows). The green arrow shows polarization sensitivity, which will be discussed in greater detail in the following sections. Masson's Trichrome histology (bottom) confirms OCT imaging, with clearly defined bone and cartilage. Image courtesy of Hermann et al. [6].

**Figure 3:**
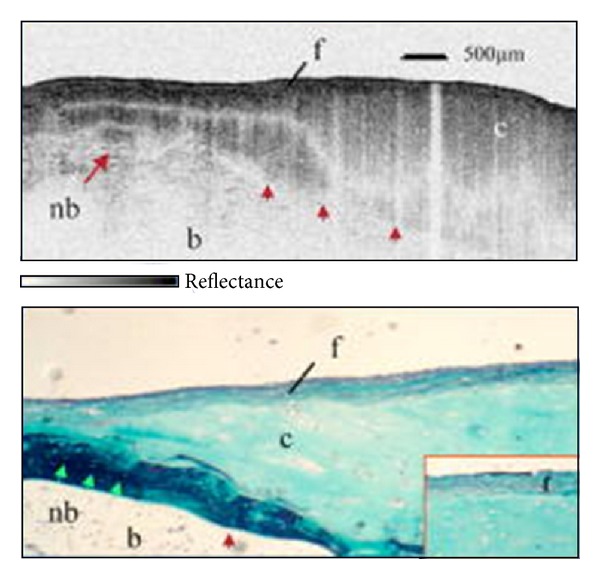
OCT of severely osteoarthritic cartilage. OCT (top) shows bone (b) and cartilage (c) with heavy cartilage loss on the left. The previously clearly defined cartilage/bone interface is destroyed, with a pathologic fibrous band on top (red arrows) and new bone growth (nb). Masson's Trichrome (bottom) again confirms OCT, with cartilage (c), bone (b), new bone growth (nb), and a fibrous band (red arrow). Image courtesy of Hermann et al. [6].

In the initial years, extensive analysis was performed to compare and match OCT imaging with histopathology of several tissue types to confidently make distinctions between normal and diseased tissue states. With an increasing library of data and literacy of the OCT images, pathological states became confidently identified in the imaging. General microstructural features are easily distinguished, such as the thickness of cartilage and subchondral bone and the presence of pathological fibrocartilage. This can be seen in Figures 2 and 3. OCT has shown great promise in evaluating human OA. It can assess the extent of OA progression, as well as the efficacy of both potential chondroprotective agents and surgical techniques.

Polarization-sensitive OCT (PS-OCT) imaging is particularly attractive in osteoarthritis detection. It is capable of assessing both the general structure and collagen organization of the most relevant tissue types, including cartilage and tendon. Birefringence, used to assess collagen organization, is a characteristic of tissues with highly organized structures, which decompose and modify the polarization state of incident light. This birefringence reflects the degree of microstructure organization in tissue containing birefringent components such as collagen, as the disruption and loss of collagen in many pathological processes results in the loss of polarization sensitivity [6, 12, 14, 40–44]. This is crucial to assess the extent of OA, as the disruption of highly organized tissue, particularly collagen, is an initial marker of OA.

**Figure 4:**
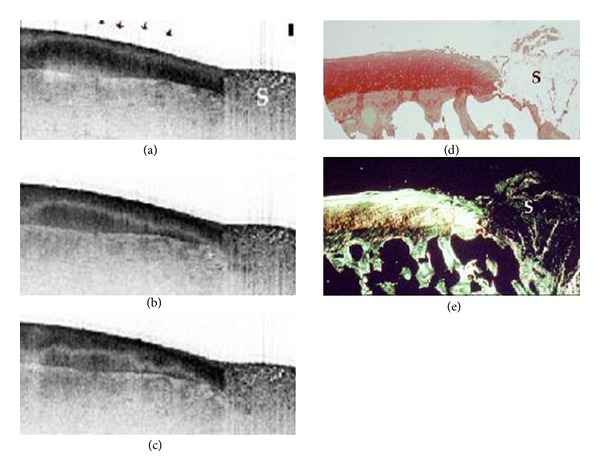
PS-OCT sensitivity of normal cartilage. (a)–(c) OCT image of cartilage at three different polarization states. They depict well defined bands that shift homogeneously with the change in polarization states, which is indicative of organized collagen. (e) Picrosirius red stain of the tissue that shows a homogeneously bright orange/yellow articular cartilage suggesting strongly organized collagen. Image courtesy of Drexler et al. [7].

**Figure 5:**
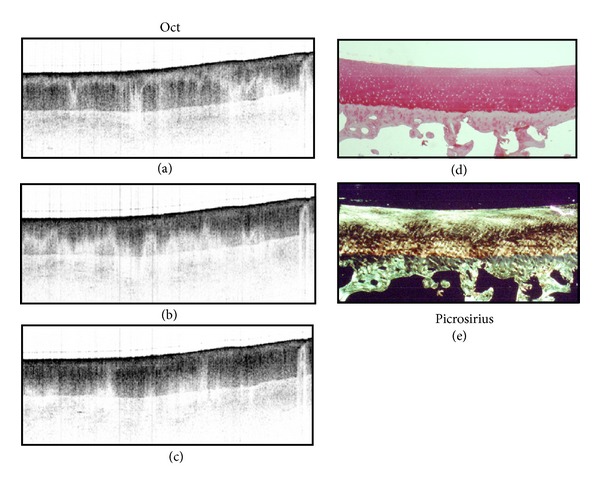
PS-OCT imaging of mildly disease cartilage. (a)–(c) OCT imaging illustrating a banding pattern that is not as defined with areas of signal dropout and does not move homogenously with changes of polarization state. (e) Picrosirius red stain of the tissue which shows some areas of organized collagen but has areas where intensity of the stain is lacking. This lack of homogeneity suggests mild disease. Image courtesy of Drexler et al. [7].

Normal human cartilage imaged by PS-OCT is presented in Figure 4 [6, 12, 40, 41, 45]. PS-OCT imaging (Figures 4(a)–4(c)) shows the positions of the bands change with the manipulation of the incident light polarization state, moving with a homogeneous rate across the sample without signal intensity dropout. This characteristic is attributed to the highly organized-collagen, which gives the cartilaginous tissue birefringent properties, and is verified with histopathology (Figure 4(e)). The bright yellow homogeneous picrosirius staining represents highly organized type II collagen-laden tissue, confirming the absence of OA. In contrast to Figure 4, Figure 5 demonstrates the initial loss of organized collagen, indicating initial disease progression [40]. Comparing this PS-OCT imaging in a mildly diseased cartilage sample (Figures 5(a)–5(c)) with Figure 4, a banding pattern is seen that shifts as the polarization state is manipulated, but does not move together in a linear fashion. These areas of signal intensity dropout and banding pattern nonuniformity are one of the first markers for the loss of parallel structure, decreased striation, and irregular shape of the collagen structure. Again confirmed with histopathology (Figure 5(e)), areas of collagen loss are represented by the loss of the intense yellow hue present in the previous histological sample. In both Figures 2 and 3, the hematoxylin and eosin stains show a smooth, homogeneous surface with full thickness and without lacunar proliferation, representing a visually grossly normal articular surface cross-section. This ability of PS-OCT to produce high-resolution optical biopsies of the tissue microstructure makes this imaging modality tremendously promising in the assessment of early OA. It is also performed in real time.

**Figure 6:**
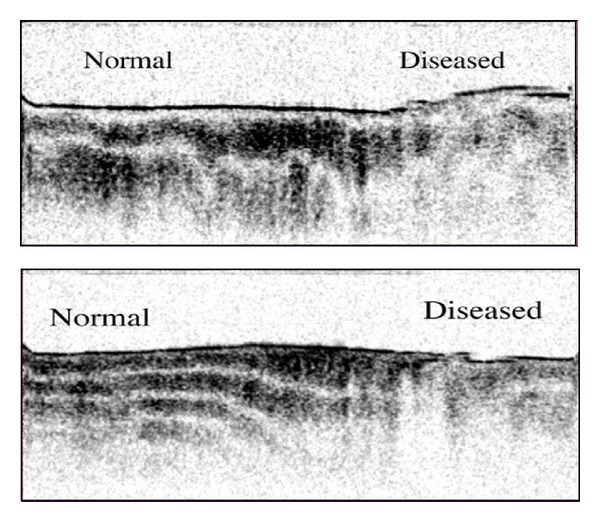
In vivo imaging of human knee cartilage. The left of the image is relatively normal cartilage, by the banding pattern, while the right is more significantly diseased and has lost polarization sensitivity. Image courtesy Li et al. [8].

Figure 6 shows normal and pathologic polarization changes in vivo [8]. This image series of in vivo human knee cartilage shows a banding pattern discrepancy between the left and right sides; bands become less homogeneous with large areas of signal intensity dropout as you move right. The far right not only shows loss of homogeneous and tight banding patterns but also shows a nonuniform articular surface. This loss of birefringence, or polarization sensitivity, again represents an early marker of OA. Note that normal tissue and diseased tissue are directly adjacent to each other and how the scanning beam of PS-OCT immediately captures the discrepancy in a single imaging frame. This capability of assessing early disease has great potential for early interventions.

**Figure 7:**
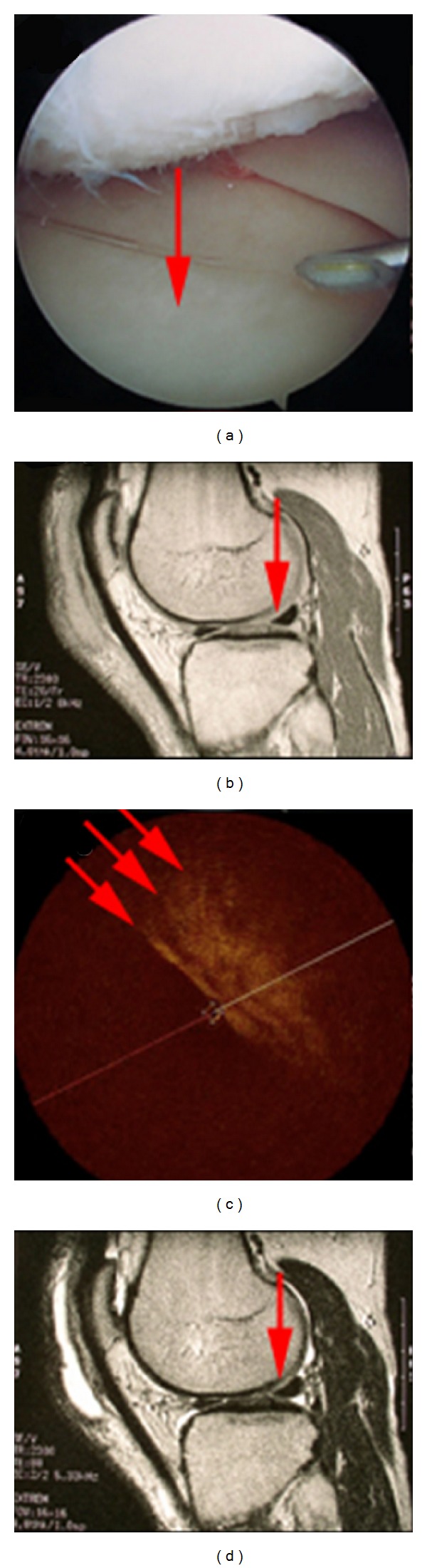
(a) Arthroscopic image of normal medial tibial cartilage. The red arrow represents a section of smooth normal looking cartilage. (b) and (d) MR image of the joint using T1 and T2 (top to bottom) relaxation states. The sagittal cross-section shows a uniformly homogeneous articular layer at the indicated point. (c) OCT image of area represented by the arthroscopic image. The OCT image shows a uniform banding pattern, indicative of healthy cartilage. Image courtesy of Zheng et al. [9].

**Figure 8:**
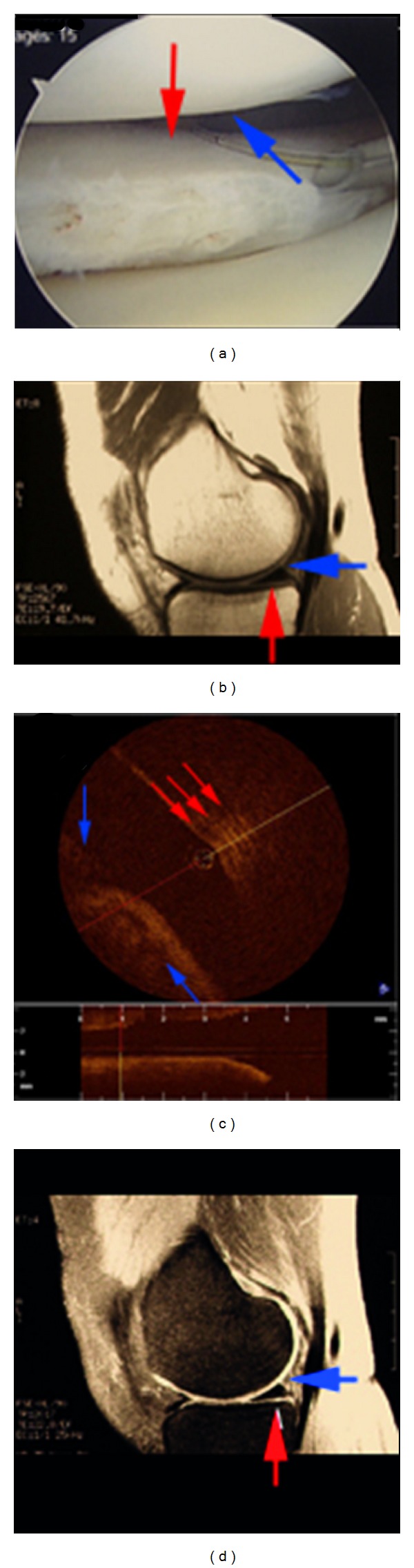
(a) Arthroscopic image of normal meniscal cartilage and mildly diseased femoral cartilage. The red arrow represents a section of meniscus, and the blue arrow represents a section of mildly diseased cartilage. Both of these positions look normal from the arthroscopic image. (b) MR image of the joint using a T1 and T2 (top to bottom) relaxation states. The sagittal cross-section shows a heterogeneous articular layer at the blue arrow and a normal meniscus at the red arrow. (c) OCT image of area represented by the arthroscopic image. The OCT image shows a relative loss of a uniformed banding pattern at the blue arrow indicative of collagen breakdown in the cartilage yet shows a tight uniformed banding pattern at the red arrow indicative of healthy highly organized meniscus. Image courtesy of Zheng et al. [9].

An OCT endocatheter has recently been developed, which can be introduced into the human knee joint while patients undergo knee arthroscopy through an 18-gauge spinal needle port [9, 17]. The approach itself is minimally invasive and adds little additional risk to the patient.  Figure 7 represents normal articular cartilage imaged with OCT, arthroscopy, and MR images [9]. The arthroscopic image (Figure 7(a)) of the tibial plateau displays smooth uniform articular cartilage despite being directly adjacent to a feathered femoral condyle. The arthroscopic image is verified by MRI (Figure 7(b)), showing thick uniformly grey cartilage without signal dropout. The OCT image (Figure 7(c)) shows the endocatheter placed directly in the center (*z* plane) and shows three uniform tight banding patterns, indicative of highly organized collagen. Conversely, Figure 8 shows smooth uniform articular cartilage arthroscopically and shows a thick uniform band of grey cartilage without areas of dropout by MRI [9]. However, in the OCT image (Figure 8(c)) there is little evidence of a uniform banding pattern and a large area of dropout. This depicts the advantage of OCT's micron-scale resolution over other diagnostic imaging modalities; both the arthroscopic and MR images were unable to detect disorganization in the cartilage, as compared to the OCT scans, which showed early disruption. In addition, this figure shows that as the articular surface was being assessed, the meniscus could be visualized in the interim, also showing a tight uniform banding pattern indicative of highly organized tissue (birefringence). 

### 3.2. OCT Imaging with Periarticular Structures

OCT is also capable of assessing periarticular structures. One prominent example is rotator cuff issues, which affect approximately 30% of the US population, and which yields 4.5 million clinical visits and 40,000 corrective surgeries per year [3]. Acute insults result in pain with movement, reduced range of motion, and joint weakness [46–49]. Additionally, changes in the shoulder articular surface and bony spurs from the acromion can cause rotator cuff tendon (RCT) impingement. These mechanisms cause collagen fibers to become abnormal through the loss of their organized, parallel structure, decreased striation, and irregular shape [10]. Impingement syndrome, in addition to traumatic mechanical insults to the shoulder, is also responsible for full- or partial-thickness tears of the rotator cuff tendons, commonly the supraspinatus, at the enthesis point with the greater humoral tuberosity [50–52]. Even with rotator cuff repairs, RCR, 25–60% of repaired shoulders rerupture within two years [53, 54]. 

Currently, RCT injuries are repaired surgically by resection of the torn segment, flattening of the greater tuberosity, and securing of the healthy segment with anchored sutures [55]. This site becomes the new enthesis point of fibrocartilage, depending heavily on local chondrocytes, fibroblasts, and pluripotent mesenchymal cells [56–58]. Even though this anabolic process is integral to enthesis stability, the integrity of the repaired tendon cannot be underestimated in the formation of a strong enthesis. It is hypothesized that reattachment of a microstructurally damaged tendon can increase the probability of RCR failure [59]. Surgeons currently rely on experience and visual identification of injured RCT for RCR surgeries. Knowing that the attachment of structurally unstable tendon with damaged collagen microstructure is detrimental to the success of RCR, surgical assistance to correctly identify and resect damaged tendon increases the hope of successful RCR by attachment of organized tendon to the enthesis. 

**Figure 9:**
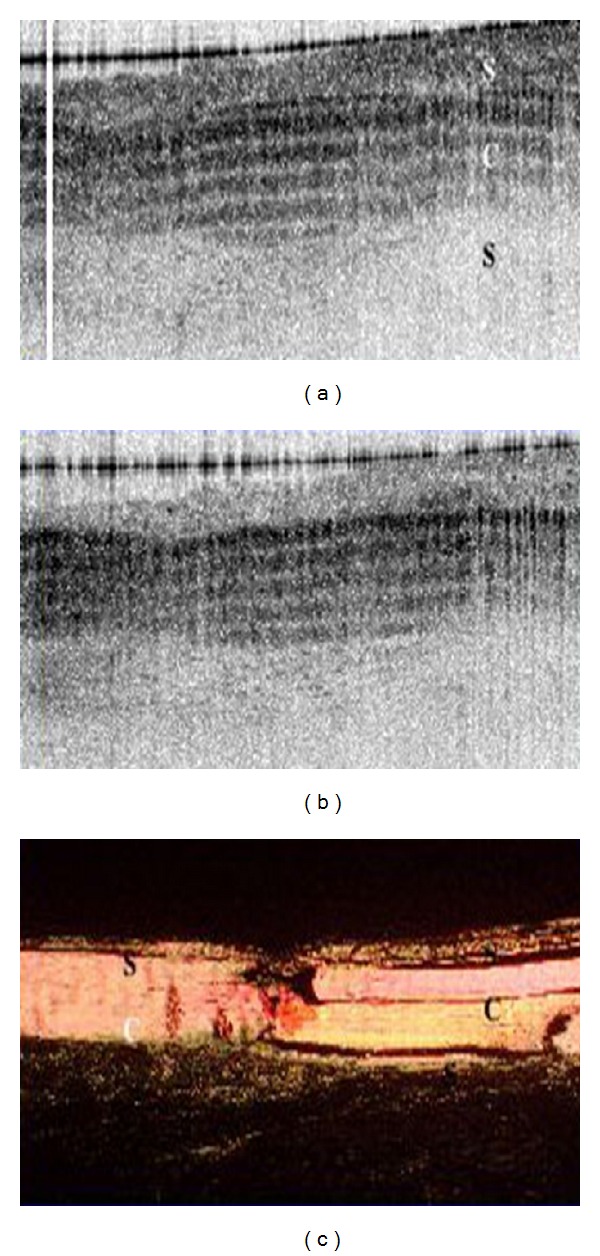
PS-OCT imaging of biceps tendon. The OCT images (top) are of biceps tendons imaged at two different polarization states. OCT effectively demarcates the layer of the tendon. The banding pattern is from the polarization sensitivity that changes with the manipulation of the polarization state, which is indicative of highly organized collagen. The picrosirius red stained sample (lower) demonstrates organized collagen by staining with intense yellow/orange color. Image courtesy of Martin et al. [10].

**Figure 10:**
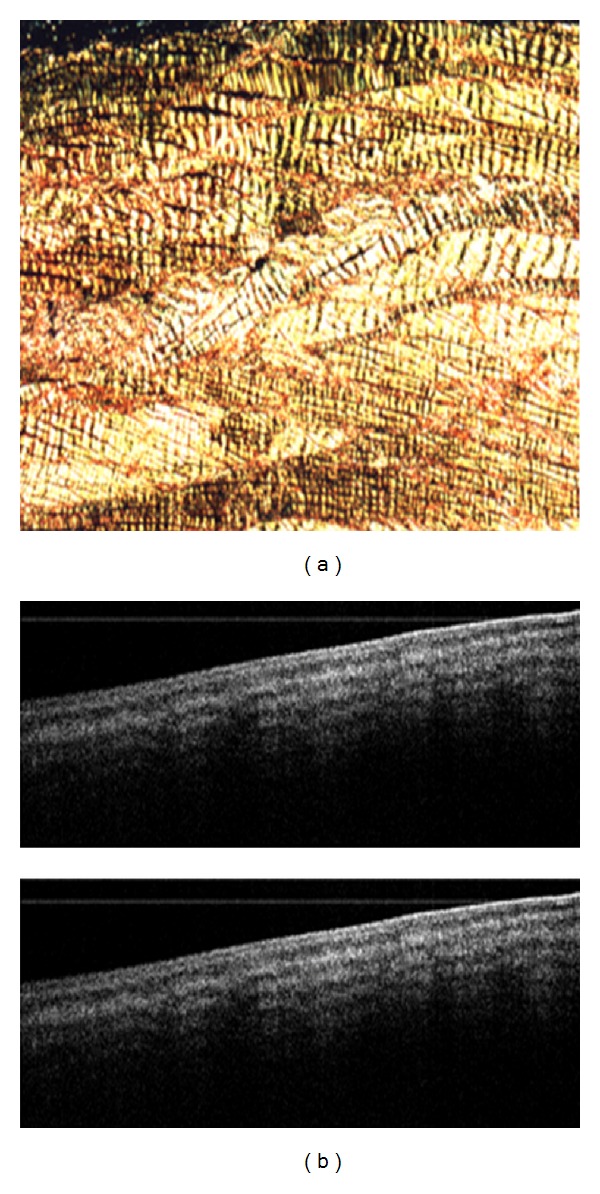
PS-OCT image of bovine meniscus with corresponding histopathology. The OCT images (a) at different polarization states shows a tight and homogenous banding pattern that is sensitive to changes in the polarization state, which is indicative of highly organized collagen. The picrosirius stained sample (b) depicts a sample with intense collagen content due to the intense yellow/orange and thick collagen bands. (Unpublished results.)

Similar to articular cartilage and meniscus, tendons and ligaments have a highly organized collagen structure and share similar properties of birefringence. Initial research in OCT imaging of periarticular structures was performed in postmortem and postsurgical resection samples [10]. Figures 9 and 10 represent periarticular tissue that shows normal structure by OCT and histopathology and has been used as a standard for normal tissue by OCT. It is important to visualize normal tissue as a control against which to assess diseased tissue, due to tissue preservation efforts common in surgical practice. Real-time high-resolution optical biopsies with OCT allow greater balance between tissue preservation and diseased tissue resection.

Biceps tendon is another extremely birefringent tissue, which serves as a prime example of periarticular OCT imaging (Figure 9). OCT effectively demarcates the layer of the tendon, and the tight uniform banding pattern produced by the change in polarization state signifies a dense concentration of organized collagen.The sensitivity of the imaging also illustrates the difference between the fascial layer surrounding the tendon, which shows no banding pattern. This is verified by corresponding picrosirius red histopathology, which shows highly organized, thick bands of collagen [10]. Bovine meniscus, which nearly identically resembles human tissue, represents the completion of periarticular tissue (Figure 10). The OCT images taken at two polarization states show a tight banding pattern without areas of dropout, again verified with histopathology. Similarly, ligaments and other tendons have been shown to exhibit this same organized structure. Both healthy anterior cruciate ligaments and Achilles tendon display a uniform, heterogeneous banding pattern that is verifiable with histology [10, 60, 61]. Even though these varying tissues reside in different compartments within the body, they all have a similar composition and similar content of organized collagen, thus exhibiting similar birefringence and serving as controls against which to assess diseased tissue. 

**Figure 11:**
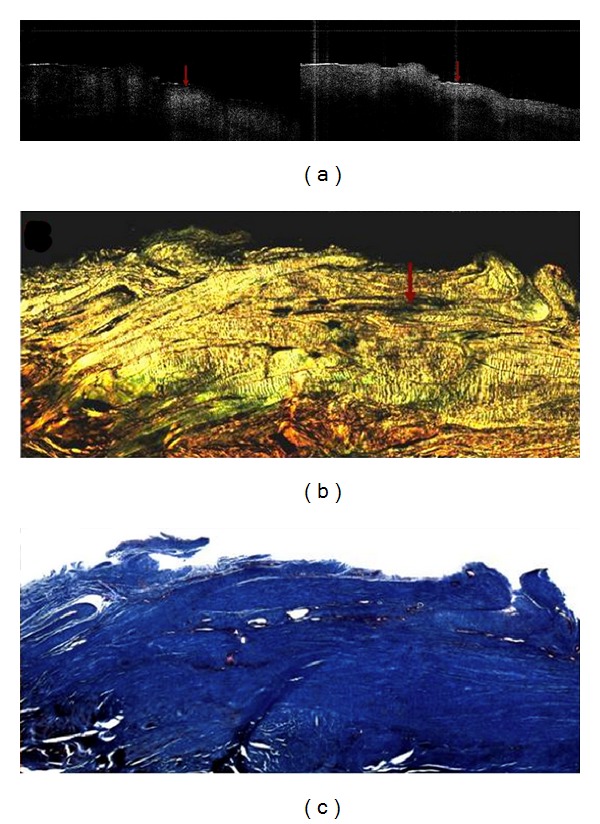
This figure demonstrates a very mildly diseased section of supraspinatus tendon. Even though this sample was considered to be “normal” by the surgeon, the picrosirius red-stained section shows a dropout of organized collagen (arrow in (b)) in otherwise healthy collagen. The trichrome section (c) shows the area is fibrocartilage. In the OCT images, back-reflection intensity changes dramatically between the two images, except the area with the arrow that essentially has no change (not birefringent) [11].

The human rotator cuff defines a complex balance of muscle, tendon, and capsule to provide an outstanding range of motion. Once this unit is damaged, it presents as a difficult task to repair and regain an acceptable range of physiological mobility and strength. This difficulty is expressed in the staggering number of failed RCRs within two years of repair. The failure point is generally at the enthesis of the tendon and bone. Although partly dependent on the local chondrocytes, fibroblasts, and mesenchymal cells, failure also depends on the quality of the organizational structure of the attached RCT [50, 51, 54]. A section of RCT taken from an RCR surgery that was deemed normal for reattachment by the surgeon can be seen in Figure 11. Comparing the OCT images with the previous figures (Figures 9 and 10), it is evident that the tissue does not have the same dense uniform banding pattern, illustrating a weaker birefringence and organizational structure. However, this sample still shows some sensitivity to changes in polarization state, signifying only a mild disease, with the region that does not change with manipulations of polarization state representing a more advanced disease. Examining the picrosirius red histology (Figure 11(b)) a section of organized collagen dropout at the arrow is otherwise surrounded by healthy collagen-laden tendon, mirroring the OCT images. The trichrome stained histology shows areas of fibrocartilage at the dropout area, further explaining the lack of organization by OCT [11]. This pilot data illustrates the feasibility for PS-OCT to be used as an interoperative imaging system to assess the borderline between tendon optimal for reattachment and disrupted tendon.

### 3.3. OCT in Rheumatoid Arthritis

Rheumatoid arthritis (RA) can be considered a more aggressive global inflammatory arthritis due to the accelerated pathogenesis caused by genetic and immunological cofactors. RA affects 1% of the population, and even though some patients have mild symptoms of joint swelling/discomfort and live with little disability, more than 50% of patients with RA have aggressive disease presentation, causing great disability with substantial joint deformity and global panarticular damage after only a few years [62–66]. Treatment for this disease was previously palliative, but with the onset and popularity of disease modifying antirheumatic drugs (DMARDs) which target TNF-alpha, T-cell costimulation, B-cells, or IL-6 pathways, there is a new push for aggressive early treatment to prevent articular and periarticular damage with these medications [65, 66]. However, the field is plagued by the fact that there are no reliable markers for differentiating patient populations that need aggressive therapy at early stages from those that require less aggressive treatment for mild symptoms. Both adult and pediatric patients with RA are likely to benefit from early diagnosis by a comprehensive understanding of RA pathogenesis/biomarkers. Early detection and enhanced understanding of disease states would assist practitioners with the ability to better choose therapies and limit the disease course. RA deserves investigation with OCT, since it has shown great potential in illustrating the early markers for OA. 

**Figure 12:**
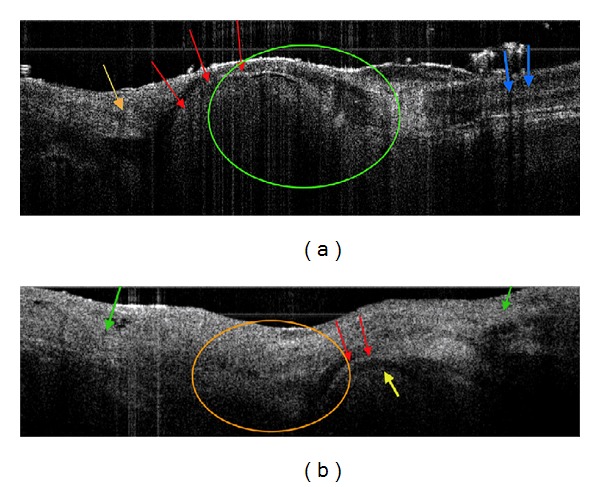
The figure depicts preliminary data of a mouse tibiotalar joint without CIA induction (a) and one at 1 week after RA induction (b). Both images have the capsule and soft tissue intact (imaged from outside the joint) generated for this proposal. The skin was removed to simulate subcutaneous imaging as a potential approach. In (a), no distorted architecture is seen. The red arrow is the cartilage surface with the synovial space above (black, minimal signal). The black gap just below the cartilage is subchondral bone. The green circle is a large synovial space and the blue a tendon. The banding in the tendon is from the highly organized collagen. The orange arrow is synovium. In Figure 3(b), 1 wk after CIA, the orange circle is synovial hypertrophy and pannus formation. Note the increased back-reflection intensity relative to (a), consistent with cellular infiltration. The red arrow is thickened cartilage (swollen in early arthritis). The subchondral bone (yellow arrow) is wider with more diffuse bone below it (edema). The green arrow is soft tissue swelling outside the joint. (Unpublished results.)

Preliminary data using OCT to visualize inflammatory arthritis has been promising in animals. Preliminary images of a mouse tibiotalar joint one week after induction of collagen-induced RA and a normal mouse tibiotalus joint have shown promise (Figure 12). Both images have the capsule and soft tissue intact. The top image shows no distortion of the tissue architecture, with the articular surface and a thin synovial space indicated. The black gap just below the cartilage represents the subchondral bone, the green circle represents the synovial space, and blue arrows represent tendon, exhibiting a banding pattern signifying highly organized collagen. In contrast, the bottom image represents a mouse tibiotalus joint after one week of collagen-induced arthritis, showing synovial hypertrophy and pannus formation (orange circle) due to noted increase of back-reflection intensity relative to the top image, consistent with cellular infiltration. Cartilaginous thickening (red arrow), subchondral bone widening (yellow arrow), and more diffuse bone edema and soft tissue swelling seen outside the joint (green arrow) all point to early signs of inflammatory arthritis. This preliminary data shows the feasibility of OCT as a technology used to visualize inflammatory arthritis in hopes of tracking the progress of future pharmaceutical management of the disease and to better the understanding of early signs/markers of inflammatory arthritis.

### 3.4. OCT in Animal Models for Research

Animal models are crucial for any research destined for human use. While many animal models exist for the investigation of OA, they show many limitations, including the need to sacrifice the animals at certain time points for histology assessment. Since OCT has shown strong in vivo correlations with histology in birefringent tissue, it can be used to serially assess the same animal and follow sequential changes. OCT has recently been used in various animal models to assess normal and diseased tissue, focusing on OA, RCT, and RA.

**Figure 13:**
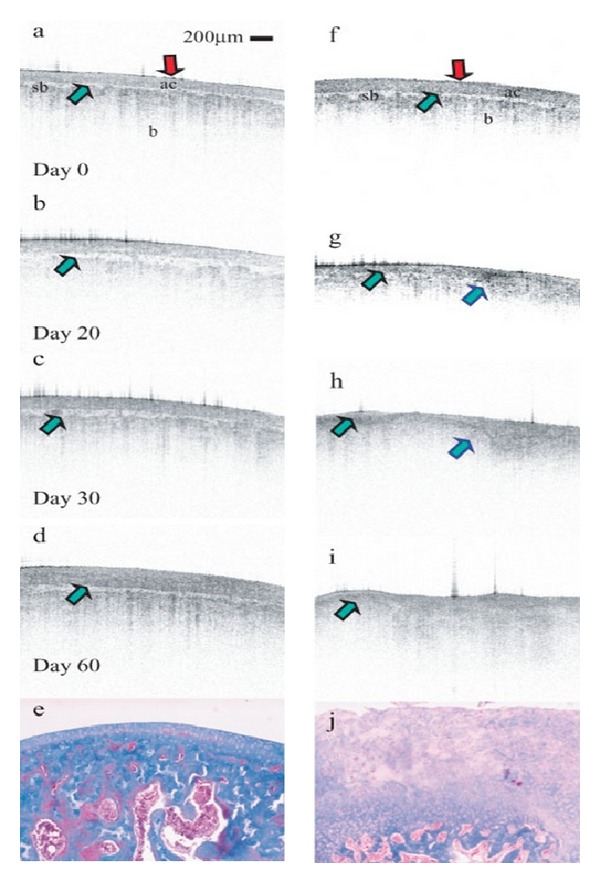
OCT imaging and corresponding histology of rat knee. Control knee (a)–(e) and diseased knee (f)–(j). Image courtesy of Adams et al. [12].

The ability to perform serial assessments of diseased tissue not only allows better understanding of the disease state, but also establishes a method to track the efficacy of therapeutics. Following the same tissue sample at multiple time points with a higher resolution than other clinical imaging modalities allows the accuracy of OCT to have a more powerful use in research. OCT images of animals with and without induced OA in the femoral condyle illustrate this ability (Figure 13) [12]. The most obvious difference between the images is the lack of cartilage on samples with induced OA. The respective histopathology confirms the lack of cartilage in the diseased joint and the presence of organized collagen in the control knee. In four papers, our lab has taken serial images of the rat knee joint with mechanically induced OA and has been able to track the progression of the disease against therapeutic treatment. The progression of inflammatory arthritis, RA, produces images different from OA, which is evident when comparing Figures 12 and 13. Much more evident are the global inflammatory/edematous markers than loss of cartilage thickness and organization in focal areas.

**Figure 14:**
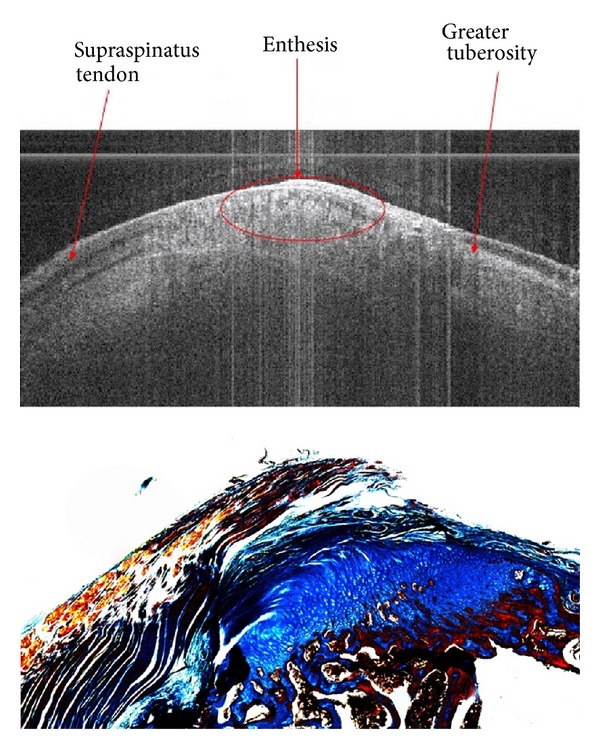
The supraspinatus tendon, enthesis, and humerus are well defined. The banding in the tendon is due to polarization rotation caused by healthy collagen which would have been previously lost in diseased areas. For histological comparison, this sample was stained with both Masson's Trichrome, to expose any structural abnormalities, and picrosirius red, to determine collagen content using a polarization filter. OCT studies in the musculoskeletal system of animals and in vivo in humans offer the potential of understanding mechanisms and identifying new therapeutic approaches, of which this image is an example. Image courtesy of Vercollone et al. [13].

Even though tracking a disease state is integral to its understanding, an animal model to assess the potential surgical techniques is just as critical. In Figure 14, the resolution of OCT can be seen in coronal image of a rat rotator cuff with corresponding trichrome histopathology [62]. The supraspinatus tendon, greater humoral tuberosity, and enthesis are well defined, and the banding in the tendon is attributed to the organization of the tissue's collagen structure. This banding and birefringence would be lost or disrupted in a diseased or torn tendon. The histology shows an intact section and tendon-enthesis junction, once again verifying OCT findings. The addition of OCT to RCT, OA, and RA animal models will allow new therapeutic approaches, as a technique to visualize the progress and understand the underlying mechanisms of diseases.

## 4. Conclusion

OCT is a potentially powerful technology for assessing musculoskeletal disease at a micron scale resolutions and in real time. More clinical trials and the increased use of adjuvant approaches are needed for it to become a routine tool for clinical assessments.

## Appendices

### A. Polarization-Sensitive OCT

PS-OCT measures the birefringence of samples by assessing the polarization state of back-reflected light. Highly organized tissue, such as collagen, modifies the polarization state of incident light in a homogeneous fashion, producing a uniform banding pattern on OCT imaging. This not only provides microstructural information from conventional OCT, but also allows the assessment of the sample's organization (in terms of degree of birefringence), which is indicative of disease progression [6, 12, 14, 40–45].

### B. Doppler OCT

Doppler OCT combines traditional OCT with laser Doppler velocimetry. As the Doppler frequency shifts do not produce a large effect on the conventional OCT image, they can be used concurrently. And, as the wavelength of light is shorter than that of sound, the resolution is far greater than that generated by traditional ultrasound Doppler imaging. Adding this high-resolution spatial imaging to the traditionally non-imaging technique of laser Doppler velocimetry allows for the assessment of clinical flow measurements, such as blood flow [14].

### C. OCT Elastography

This technique measures the mechanical properties of tissue in a manner similar to ultrasound elastography. A mechanical force is applied to a tissue sample, and the resultant alteration in the tissue is recorded, producing information about the tissue's composition. Applications include phantoms and skin, with recent advances in the use of Air-Indentation OCT Elastography to assess mechanical properties of in vivo human skin in the extremities [67–69].

### D. OCT Spectroscopy

In addition to OCT absorption spectroscopy, which functions by combining traditional OCT with spectroscopic analyses, recent work has shown promise in the development of a second-order correlation (SOC) measurement technique. This technique utilizes photon entanglement, a quantum phenomenon which gives insight into tissue composition [70, 71].
